# Public health strategy vs. golden standard for ocular cancer care in Brazil

**DOI:** 10.5935/0004-2749.20200021

**Published:** 2020

**Authors:** Rubens Belfort Neto

**Affiliations:** 1 Departamento de Oftalmologia, Escola Paulista de Medicina, Universidade Federal de São Paulo, São Paulo, SP, Brazil

Late treatment of ocular cancer is a national health problem in developing countries, including Brazil. Late treatment is associated with great patient suffering, financial costs, morbidity, and mortality; not to mention high public expenditure. In an ideal world, all patients with ocular cancer would be treated by well-trained and experienced ocular cancer specialists in a timely manner without undue burden to the patients. In an attempt to achieve this in Brazil, we have assembled a team of onco-ophthalmologists from Escola Paulista de Medicina to empower general ophthalmologists to diagnose and treat less complex ocular cancers, while guiding the referral of complex cases to onco-ophthalmologists.

Our solution uses the multiplatform message app, WhatsApp, as part of a system called “OncoPhone,” which allows local general ophthalmologists to send us case details with images and to get quick responses from our group of specialists. In this way, we can improve both the diagnoses and treatment of ocular cancers. In addition, we will publish videos to YouTube to provide further guidance on how to treat some complex cases. Understandably, not all cases can be addressed by remote consultation; we advise general ophthalmologists to immediately refer very complex cases, such as those with retinoblastoma and advanced conjunctival melanoma, to tertiary centers.

Our telemedicine strategy increases access for patients to a trained ocular oncologist. However, it is a structure that is far from ideal because we firmly believe that every patient should be treated by well-trained and specialized doctors. Our effort was born in the setting of an underfunded public health infrastructure in Brazil, where we see ophthalmologists treating complex diseases such as melanoma and retinoblastoma without the proper training and experience. We have seen cases in which they conduct biopsies and other procedures based on guesses and mere feelings. In fact, even large “cancer hospitals” in Brazil hire general ophthalmologists to work in ocular cancer settings in a “learn-by-doing” scenario. General ophthalmologists are not considered to be adequately trained to perform a corneal transplant or macular surgery, yet they are somehow expected to take care of complex vision and life-threatening illnesses without the proper training.

Take the example of transpupillary thermotherapy (TTT) for choroidal small-to-medium-sized melanomas that was considered an efficient treatment^(1)^ and is still used by many general ophthalmologists but is now known to be a substandard treatment for almost all choroidal melanomas in the absence of brachytherapy: TTT alone leads to recurrences in 20%-50% of patients after 10 years, depending on the tumor characteristics, resulting in high chances of metastasis and death^(2)^. Moreover, studies have suggested that the laser in TTT, combined with plaque brachytherapy, does not improve tumor control and can make vision prognosis worse^(3)^. Despite the progress in the field of ocular oncology, many general ophthalmologists still lack knowledge and experience.

Thus, ocular tumors cannot continue to be treated by pseudo-specialists in large centers. International guidelines and protocols need to be followed when treating patients with ocular cancer. We hope to never see another blind eye due to a contraindicated biopsy on a patient with choroidal melanoma. We hope to never see another pediatric death due to inappropriate treatment of retinoblastoma with multiple vitrectomies and anti-VEGF injections for a “presumed Coats’ Disease.” There is no more room for ignorance. All of us should act on this issue.

Is TTT for choroidal melanoma dead? YES. As should be the substandard care provided by pseudo-ocular-oncology specialists.
